# A Spatial Analysis of Tuberculosis Related Mortality in South Africa

**DOI:** 10.3390/ijerph182211865

**Published:** 2021-11-12

**Authors:** Dan Kibuuka, Charles Mpofu, Penny Neave, Samuel Manda

**Affiliations:** 1 Planning, Funding and Outcomes Unit, Child, Youth and Women Health, Waitemata District Health Board, Auckland 0740, New Zealand; 2 School of Public Health and Psychosocial Studies, Faculty of Health and Environmental Sciences, Auckland University of Technology (AUT), Auckland 0627, New Zealand; charles.mpofu@aut.ac.nz; 3 Immunisation Advisory Centre, UniServices, The University of Auckland, Auckland 1023, New Zealand; p.neave@auckland.ac.nz; 4 Biostatistics Research Unit, South African Medical Research Council, Pretoria 0001, South Africa; Samuel.Manda@mrc.ac.za; 5 Department of Statistics, University of Pretoria, Pretoria 0028, South Africa

**Keywords:** tuberculosis, mortality, age-sex-standardised, autocorrelation, spatial analysis, hot spot analysis, South Africa

## Abstract

*Background:* South Africa, with an estimated annual tuberculosis (TB) incidence of 360,000 cases in 2019, remains one of the countries with the largest burden of TB in the world. The identification of highly burdened TB areas could support public health policy planners to optimally target resources and TB control and prevention interventions. *Objective:* To investigate the spatial epidemiology and distribution of TB mortality in South Africa in 2010 and its association with area-level poverty and HIV burden. *Methods:* The study analysed a total of 776,176 TB deaths for the period 2005–2015. Local and global and spatial clustering of TB death rates were investigated by Global and Local Moran’s Indices methods (Moran’s I). The spatial regression analysis was employed to assess the effect of poverty and HIV on TB mortality rates. *Results:* There was a significant decrease in TB mortality rate, from 179 per 100,000 population in 2005 to 60 per 100,000 population in 2015. The annual TB mortality rate was higher among males (161.5 per 100,000 male population; (95% confidence interval (CI) 132.9, 190.0) than among females (123.2 per 100,000 female population; (95% CI 95.6, 150.8)). The 35–44 age group experienced higher TB mortality rates, regardless of gender and time. Hot spot clusters of TB mortality were found in the South-Eastern parts of the country, whereas cold spot clusters were largely in the north-eastern parts. Tuberculosis death rates were positively associated with poverty, as measured by the South African Multidimension Poverty Index (SAMPI) as well TB death rates in the neighbouring districts. *Conclusion:* The findings of this study revealed a statistically significant decrease in TB deaths and a disproportionate distribution of TB deaths among certain areas and population groups in South Africa. The existence of the identified inequalities in the burden of TB deaths calls for targeted public health interventions, policies, and resources to be directed towards the most vulnerable populations in South Africa.

## 1. Introduction

Tuberculosis has been a preventable disease since 1921 when the Bacillus Calmette-Guerin (BCG) vaccine was discovered, but it still poses a major global health problem. Annually, the number of TB prevalent cases is approximately 10 million people globally [1] with more than one million deaths attributed to TB [2]. Most of these deaths are in low-income countries. South Africa remains one of the most TB burdened countries with an estimated annual TB incidence of 301,000 cases in 2018. In 2019, the World Health Organisation estimated the mortality rate of all forms of TB for South Africa, excluding deaths among those coinfected with TB/HIV, to be 37 and 73 per 100,000 population, respectively [1]. It is estimated that there is a new infection with the TB bacterium every second globally [3]. For almost a decade, TB has caused more deaths than HIV/Acquired Immunodeficiency Syndrome (HIV/AIDS), making it the single leading cause of death from an infectious disease [1]. 

South Africa is one of the most unequal countries in the world with respect to income variation with a large proportion of South Africans who are affected by extreme poverty. Almost half (45%) of the South African population is still living on less than USD 2 per day. Poverty has affected the health of most South Africans predominantly due to an inability to access the basic requirements of life; for instance, sufficient nutrition, adequate sanitation, and appropriate housing conditions [4,5,6,7]. Household crowding, usually because of poverty, has been related to diseases of the respiratory system such as TB [8]. South Africa has one of the world’s worst TB epidemics, which is fueled by HIV [9]. It has been shown that for HIV-infected individuals with clinically symptomatic TB, the risk of death is about three to seven-fold higher than in those individuals who are infected with TB but are HIV negative [10,11], whereas in those individuals with AIDS, developing TB will increase the overall mortality by one-third [12].

Therefore, this research aims to contribute to TB control programs in South Africa by showing the value of understanding the spatial distribution of TB deaths in South Africa. There is a lack of previous research papers on TB spatial analysis and regression. Furthermore, the study aims to confirm an association between TB, Social Economic Status (SES) (using the South African Multidimensional Poverty Index), and HIV. 

## 2. Materials and Methods

### 2.1. Data Sources

This research used secondary data from four data sources, namely, mortality and causes of death in South Africa from Statistics South Africa, the South African Multidimensional Poverty Index (SAMPI) from Statistics South Africa, HIV data from the South African national Department of Health, and census and mid-year population estimates from Statistics South Africa. In South Africa, national mortality statistics are captured in the civil registration system. In recent years, the country has adopted the Africa Programme on Accelerated Improvement of Civil Registration and Vital Statistics (APAI-CRVS) [13]. The mortality data are based on death notification forms (Form BI-1663) that are submitted to the Department of Home Affairs offices for death registration as a requirement by the country’s Births and Deaths Registration Act No 51 of 1992 [14]. We extracted data on TB deaths from the civil registration system in South Africa. We used ICD-10 codes A16-A19 for TB deaths. 

SAMPI was constructed by Statistics South Africa to profile poverty at a household level using variables from censuses from the years 2001 and 2011. The SAMPI score is derived from the product of the headcount, and the intensity of the poverty experienced by households [15]. The antenatal HIV prevalence data for 2010 were obtained from the national Department of Health of South Africa.

### 2.2. Methods of Data Analysis

We first conducted a descriptive epidemiological analysis of TB deaths for the period 2005–2015 with respect to gender, age group, provinces, and district municipalities in South Africa. This was followed by a spatial autocorrelation analysis and a spatial lag regression (also known as the spatial autoregressive (SAR)) model of TB deaths for 2010. Spatial autocorrelation was conducted to establish the degree of similarity between TB death rates in a district municipality to TB death rates in neighbouring district municipalities. We conducted a spatial regression analysis (SAR) to investigate a potential association in the spatial variations of TB deaths (dependent variable), in instances where there were hot spot clusters of district municipalities. The independent variables for this study were the SAMPI and HIV.

To enable comparison of TB mortality for population groups that may have a different age structure, as may be the case for South Africa’s district municipalities, age-sex-standardised rates were computed. The direct standardisation technique was used to derive the age-sex-standardised TB death rates using the age distribution of the 2011 population census as the standard age structure.

The presence of spatial autocorrelation was determined using both Global and Local Moran’s Indices methods (Moran’s I) [16]. Suppose that $Y_{1},\ldots ,Y_{52}$ are observations of the TB mortality rates for the n=52 districts in South Africa for the year 2010. We also have $W\;=\left[w_{ij}\right]$ as an adjacency matrix, which quantifies the connection between the districts. Global Moran’s I is defined as follows:

(1) $$I=\frac{n}{W_{0}}\times \frac{\sum_{i}^{n}\sum_{j}^{n}w_{ij}\left(Y_{i}-\bar{Y}\right)\left(Y_{j}-\bar{Y}\right)}{\sum_{i=1}^{n}{\left(Y_{i}-\bar{Y}\right)}^{2}},i,\;j=1,\ldots ,52$$

where $\bar{Y}=n^{-1}\sum_{i}^{n}Y_{i}$ and $W_{0}=\sum_{i}^{n}\sum_{j}^{n}w_{ij}$. One approach is to define a neighboring district would be when it shares a boundary (i.e., contiguous districts) or it is within a certain distance or it100 km) or it is among the nearest district (e.g., the 3 closest districts). For this study, we adopt the first definition where we take $w_{ij}=1$ if the districts share a boundary, otherwise it is 0. By its construction, the Global Moran’s I varies from −1 to +1, where positive values would mean that the districts close together had similar TB mortality rates, while negative values would means that districts close together had more dissimilar rates than those districts further away. For local spatial dependence, the Local Moran’s $I_{i}$ for district i defined as

(2) $$I_{i}=n\times \frac{\left(Y_{i}-\bar{Y}\right)\sum_{j}^{n}w_{ij}\left(Y_{j}-\bar{Y}\right)}{\sum_{i=1}^{n}{\left(Y_{i}-\bar{Y}\right)}^{2}},\;I,\;j=1,\ldots ,52$$

A positive Local Moran’s $I_{i}$ would indicate that ditrsict i has neighboring districts with similarly high or low TB mortality rates.

To model the incident of TB related mortality using predictors SAMPI and HIV, the spatial lag regression (also known as the spatial autoregressive (SAR)) model was used. The SAR model accounts for dependence between TB deaths data in a municipality and neighboring municipalities’ TB mortality data. Under the normality assumption on Y, the spatial lag model is defined as [16].

(3) $$Y_{i}=\rho \overset{n}{\underset{n}{\sum }}w_{ij}+X_{i}\beta +\varepsilon$$

where $\varepsilon \sim MVN\left(0,\sigma^{2}I_{52\times 52}\right)$, ρ is the spatial lag parameter which measures the strength of spatial dependence in the TB death rates, vector $X_{i}\left(j=1,2\right)$ is covariate vector consisting of poverty, and HIV burden, and they are associated with a 2×1 vector β of regression parameters. Most GIS packages default to using row-standardized weights, with $\sum_{\mathrm{j=1}}^{n}w_{ij}=1$, in which the spatially lagged predictor would be the average of the TB mortality rates of neighboring districts. Thus, the SAR model adds a spatially averaged vector as a covariate, reflecting the TB mortality data from the neighbouring districts to aid in explaining the variation in the TB death rates between the districts. Spatial analysis was performed using the Geographic Data Analysis (GeoDa) version 1.14. 

## 3. Results

The mean annual TB death rate for the study period was 142.2 per 100,000 population, 95% CI [114.2, 170.2]. Overall, there was a statistically significant decrease in TB deaths of less than 1 TB death (0.01) per 100 000 population annually at 5% significance (F (1,10) = 93.58, *p* = 0.001) for the period 2005–2015. The mean annual TB death rates for males and females were 161.5 per 100,000 population, 95% CI [132.9, 190.0] and 123.2 per 100,000 population, 95% CI [95.6, 150.8], respectively. The annual TB death rate for both male and females decreased over the study period (Table 1). 

**Figure 1:**
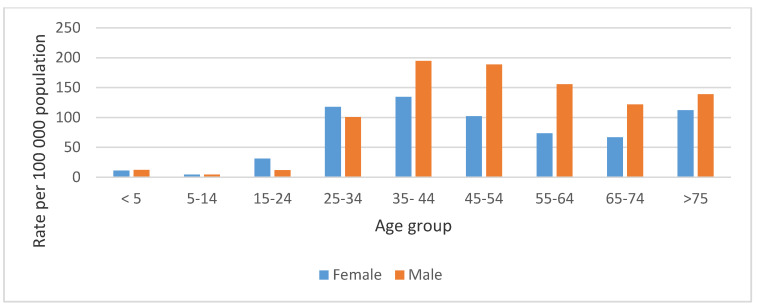
Tuberculosis mean annual death rate per 100 000 population by gender and age group in South Africa, 2005–2015.

The highest mean annual TB death rate for both males and females was recorded in the 35–44 age group, 194.7 per 100,000 population (129,724 deaths), and 134.3 per 100,000 population (89,480 deaths), respectively (Figure 1). The mean annual TB death rate was also highest in the Black African population group at 136.3 per 100,000 population, followed by the Coloured population group at 63.8 per 100,000 population for the period of study.

**Figure 2:**
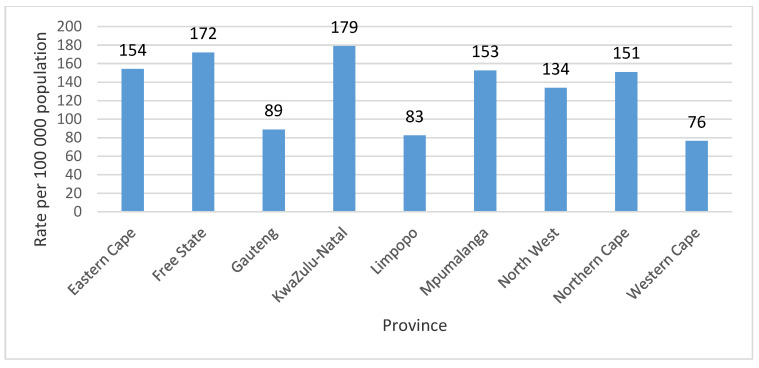
Mean annual tuberculosis death rate per 100 000 population by province in South Africa, 2005–2015.

For the study period, the mean annual TB death rate for the provinces was 132.3 per 100,000 population at 95% CI [101.9, 162.4]. The highest mean annual TB death rate for the period 2005–2015 was recorded in the KwaZulu-Natal province at 178.9 per 100,000 population, closely followed by Free State province at 172.1 per 100,000 population (53,940 TB deaths). The Western Cape province recorded the lowest mean annual TB death rate 76.5 per 100,000 population (45,513 TB deaths) for the period of study (Figure 2).

The mean annual TB death rate for the district municipalities for the study period was 155.5 per 100,000 population at 95% CI [139.6, 171.4]. Ugu district municipality had the highest mean annual TB death rate of 312.0 per 100,000 population (22,722 TB deaths); followed by Amathole district municipality 233.1 per 100,000 population (21,791 TB deaths). Vhembe and Overberg district municipalities had the lowest mean annual TB death rate 66.1 per 100,000 population (9348 TB deaths) and 66.3 per 100,000 population (1800 TB deaths), respectively, for the period of study.

### 3.1. Spatial Autocorrelation

The results for both the Moran’s I and Z values were positive and greater than zero for the overall, male, and female age-sex-standardised TB death rates. In addition, the results were significant at the 99% confidence interval. These results are indicative of a presence of autocorrelation for the overall, male, and female age-sex-standardised TB death rates. Therefore, there was significant similarity between neighbouring district municipalities for the overall, male, and female age-sex-standardised TB death rates (Table 2).

### 3.2. Local Spatial Clustering for TB Death Rates

**Figure 3:**
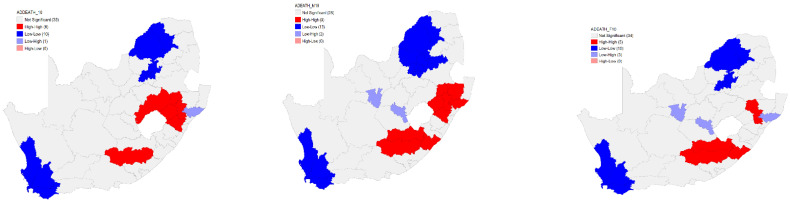
Local Moran’s I clustering of the overall (**left**), male (**middle**), and female (**right**) age-sex-standardised TB death rates by district municipality, 2010.

The Local Moran’s I analysis for clustering for the overall age-sex-standardised TB death rates revealed six high–high clusters. Ten low–low clusters for the overall age-sex-standardised TB death rates were identified. Furthermore, the Local Moran’s I analysis identified one low–high cluster. However, there were no high–low clusters that were identified for the overall age-sex-standardised TB death rates in 2010 (Figure 3).

For the male, nine high–high clusters were identified on the eastern side of the country. In addition to the high–high clusters, 13 low–low clusters for the male age-sex-standardised TB death rates were identified in 2010. Furthermore, the Local Moran’s I clustering analysis identified two low–high with no high–low clusters identified for the male age-sex-standardised TB death rates (Figure 3).

In 2010, the results of the Local Moran’s I analysis for clustering for the female age-sex-standardised TB death rates in 2010 identified five high–high clusters located on the eastern part of the country. Furthermore, the Local Moran’s I revealed a presence of 10 low–low clusters. The results further revealed a presence of three low–high clusters. There were no high–low clusters identified for the female age-sex-standardised TB death rates (Figure 3).

### 3.3. Intensity of TB Death Rates (Hot Spots)

**Figure 4:**
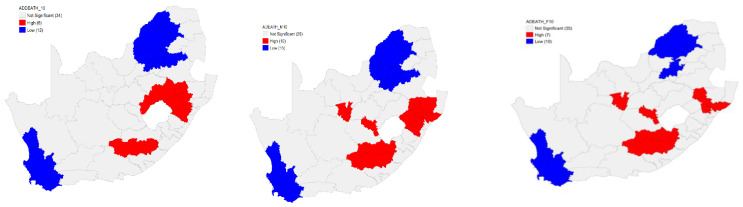
Local G hot and cold spots for the overall (**left**), male (**middle**), and female (**right**) age-sex-standardised TB death rates by district municipality, 2010.

Six hot spots were identified in South Africa by the Local G (hot spots) analysis for the overall age-sex-standardised TB death rates in 2010. These hot spots were on the eastern side of the country, located in three provinces. Twelve cold spots were identified. In other words, the results revealed a high risk of TB deaths in 2010 in the South African population on the eastern part of the country with a lower risk in the northern and southern parts (Figure 4).

The Local G hot spots analysis identified 10 hot spots for the male age-sex-standardised TB death rates. These hot spots were in the central part with the majority on the eastern side of the country. Analysis further revealed a presence of 13 cold spots in 13 district municipalities in the country. These results indicate that, in 2010, there was a high risk of TB deaths in the male in the South African population on the eastern and central parts of the country and a lower risk in the northern and southern parts (Figure 4).

There were seven hot spots for the female age-sex-standardised TB death rates identified by the Local G hot spots analysis. Many hot spots were located on the eastern side of the country. Analysis further revealed a presence of 10 cold spots in the country (Figure 4). The Local G hot spots analysis was able to identify more hot spots than the Local Moran’s for both the male and female age-sex-standardised TB death rates.

### 3.4. Spatial Regression Analysis

Spatial lag regression analysis for the overall age-sex-standardised TB death rates identified a positive association between the overall age-sex-standardised TB death rates for both HIV and SAMPI in the neighbouring district municipalities. The association between the overall age-sex-standardised TB death rates and HIV was not statistically significant at *p* value 0.49995. However, the association with SAMPI was statistically significant at *p* value 0.05887 (Table 3).

The spatial lag regression analysis for the male and female age-sex-standardised TB death rates revealed a positive association between the male and female TB death rates for both HIV and SAMPI in the neighbouring district municipalities. The association between the male age-sex-standardised TB death rates with HIV was not statistically significant at *p* value 0.73237. However, the association with SAMPI was statistically significant at *p* value 0.01727. The association between the female age-sex-standardised TB death rates and HIV and SAMPI were both not statistically significant at *p* value 0.58862 and 0.06040 for HIV and SAMPI, respectively (Table 3).

## 4. Discussion

This study set out to investigate the spatial epidemiology of TB mortality in South Africa using death notification data for the period 2005–2015. Both spatial clustering measures and spatial lag regression were used, the latter to examine the association of TB mortality rates, area-level poverty, and HIV burden. The study found a decline in the rate of TB deaths over the study period. We also found that TB death rates varied between gender and age-groups, provinces and district municipalities. Black South Africans and south-eastern parts of the country had higher TB death rates. This study revealed spatial dynamics of TB which has shown a distinct district municipality trend of hot spots occurring in areas that mostly have high levels of SAMPI and HIV. TB death rates were higher in the district that had higher levels of SAMPI.

The study showed a statistically significant decline in the rate of TB deaths over the study period. These findings were like the global trend [17] and for studies in countries such as Japan [18], the USA [19], and the UK [20]. This downward trend is an important finding, especially for the Sustainable Development Goals and the End TB strategy—both of which call for a reduction in TB deaths. The study further revealed that for the period 2005–2015, there was a higher mean annual TB death rate in males than in females. This finding is similar with other studies in other countries [18,21,22]. Although this study could not independently establish why there were more male than female TB deaths, it may be argued that it is due to risk factors associated with TB that are more prevalent in men, for example, smoking [22] and co-morbidities such as HIV [18]. Another important finding is that for the study period, for both males and females, the mean annual TB death rate was highest in the 35–44 age group. While a similar study in South Africa had the same finding [23], this finding is in contrast to the findings of a study in Japan where TB deaths were higher in the older age groups, believed to be due to increased exposure of the elderly to other chronic health conditions [18]. Tuberculosis deaths in the 35–44 age group in South Africa has implications in that this is the age group that is economically active; therefore, it is important that health workers in the TB control programme pay particular attention to this age group in order to reduce the number of TB deaths.

A previous study showed similar findings of high TB deaths in KwaZulu-Natal province [23]. Many parts of KwaZulu-Natal and Eastern Cape provinces province are rural with most inhabitants being Black Africans. According to Mayosi and colleagues, a large proportion of South Africans live in poverty with limited access to, for instance, reasonable housing conditions [4]. Moreover, it is known that there is an association between TB burden and socio economic status (SES) [24,25,26], and that the poor maybe unable to access health care services [27]. These findings mean that public health policies on TB prevention and management should directed in south-eastern parts of the country. We have also found significant evidence of spatial closuring in the TB mortality rates. This is consistent with some of the previous studies that found strong spatial autocorrelation [28,29,30]. The TB clusters could be an indication that populations in sorounding districts in share common TB risk factors [31,32]. The identification of these clusters and hot spots underscores the importance of understanding TB transmission dynamics between districts. This requires recognition of the local context in the efforts to prevent and control infectious diseases such as TB with inclusion of a neighbourhood approach, for instance, the district municipalities that are located next to each other. Our findings, are important in providing empirical evidence to the Department of Health to focus the identified high TB risk districts prioritised for TB interventions.

## 5. Study Limitations

This study used secondary TB data from the Civil registration system. This TB data source may have missing TB deaths due to the incompleteness of the Civil registration system. Despite this limitation, the study added new knowledge by investigating spatial TB deaths at a district level—a level at which primary health care services such as TB services are offered in South Africa. In addition, this study was able to identify hotspots and relate them to SES and HIV.

## 6. Conclusions

The findings revealed a significant decrease in TB deaths and a disproportionate distribution of TB deaths among certain groups of people in South Africa (Black African, males, and those in the 35–44 age group) and certain provinces and district municipalities. The findings further revealed strong spatial autocorrelation for the overall, male, and female age-sex-standardised TB death rates, thus revealing a significant similarity between neighbouring districts regarding TB death rates. In addition, the findings revealed a significant positive association between district level TB deaths and poverty profiles, as well as the effect of TB burden in nearby districts. The existence of these identified inequalities in the burden of TB deaths underscores the importance of developing targeted public health interventions and policies to be directed towards the most vulnerable populations in district municipalities with hot spots. Furthermore, enough resources should be directed into TB prevention activities at a district municipal level.

## Figures and Tables

**Table 1:** Tuberculosis death rates per 100,000 population by year and gender in South Africa, 2005–2015.

Year	2005	2006	2007	2008	2009	2010	2011	2012	2013	2014	2015
TB death rate	179	186	182	178	166	153	135	122	107	98	60
Male	196	203	199	200	189	174	156	142	126	117	74
Female	162	168	165	156	145	132	114	101	88	79	45

**Table 2:** Global Moran’s Indices, Z and *p*-values for the overall, male, and female age-sex-standardised TB death rates, 2010.

Death Rates	Moran’s I	Z Value	*p*-Value	99% CI
Overall age-sex-standardised TB death rates	0.372	4.781	0.010	(0.196, 0.549)
Male	0.431	5.000	0.001	(0.237, 0.624)
Female	0.377	4.385	0.002	(0.211, 0.543)

Note: Significant at 99% confidence.

**Table 3:** Spatial lag regression coefficient, standard error, Z and *p*-values for the overall, male, and female age-sex-standardised TB death rates, 2010.

Variable	Coefficient	Std. Error	Z Value	*p*-Value
**Overall**				
Spatial lag parameter	0.418345	0.15308	2.73285	0.00628
CONSTANT	34.9371	49.247	0.709427	0.47806
HIV	1.22123	1.80868	0.675205	0.49955
SAMPI	1262.73	668.402	1.88918	0.05887
**Male**				
Spatial lag parameter	0.47336	0.143263	3.30414	0.00095
CONSTANT	47.1193	45.256	1.04117	0.29780
HIV	0.549491	1.60684	0.34197	0.73237
SAMPI	1437.1	603.592	2.38092	0.01727
**Female**				
Spatial lag parameter	0.441217	0.149964	2.94215	0.00326
CONSTANT	38.7686	36.3154	1.06755	0.28572
HIV	0.695831	1.2866	0.540831	0.58862
SAMPI	896.103	477.189	1.87788	0.06040
